# Risk of ischemic stroke in patients with prostate cancer receiving androgen deprivation therapy in Taiwan

**DOI:** 10.1186/s12885-019-6487-2

**Published:** 2019-12-30

**Authors:** Kuang-Ming Liao, Yaw-Bin Huang, Chung-Yu Chen, Chen-Chun Kuo

**Affiliations:** 1 Department of Internal Medicine, Chi Mei Medical Center, Chiali, Taiwan; 2 School of Pharmacy, Master Program in Clinical Pharmacy, Kaohsiung Medical University, No. 100, Shihcyuan 1st Rd., Sanmin District, Kaohsiung City, 80708 Taiwan, Republic of China; 3 Department of Pharmacy, Kaohsiung Medical University Hospital, Kaohsiung, Taiwan; 4 Department of Medical Research, Kaohsiung Medical University Hospital, Kaohsiung, Taiwan

**Keywords:** Androgen deprivation therapy, Ischemic stroke, Prostate cancer

## Abstract

**Background:**

Androgen deprivation therapy (ADT) in the treatment of prostate cancer may be associated with an increased risk of thromboembolic disease. The aim of our study was to investigate the association of ADT in the treatment of prostate cancer with ischemic stroke risk.

**Methods:**

We identified individuals older than 20 years of age who were newly diagnosed with prostate cancer between January 1, 2005, and December 31, 2012. Patients who experienced ischemic stroke or transient ischemic stroke before the index date were excluded. Patients who received at least one prescription for ADT within 6 months were defined as the ADT user group. Patients who did not receive at least one prescription for ADT within 6 months were defined as the ADT nonuser group. The patients were followed until the first occurrence of one of the primary outcome measures (ischemic stroke or death) or until December 31, 2013. The primary composite outcome was the time to any cause of death or ischemic stroke.

**Results:**

There was no significant difference in the primary composite outcomes in the prostate cancer patients between the ADT user and nonuser groups. Prostate cancer patients who received ADT had a higher mortality rate than those who were not treated with ADT, and the adjusted hazard ratio was 1.907 (95% confidence interval: 1.278–2.844; *P* = 0.0016) after adjusting for age, comorbidities and comedication use.

**Conclusion:**

ADT in the treatment of prostate cancer may not be associated with an increased risk of ischemic stroke. The differences in thromboembolic effects in cardiovascular disease and ischemic stroke secondary to ADT should be further discussed and evaluated prospectively.

## Background

Androgen deprivation therapy (ADT) is the key treatment for advanced prostate cancer patients or patients with high serum prostate-specific antigen levels with intermediate or high-risk prostate cancer even without imaging findings or other evidence of disseminated disease [1, 2].

Despite the frequently dramatic potential benefits after treatment, ADT has side effects that can greatly impact a patient’s quality of life and may necessitate changing the treatment strategy. These side effects include sexual dysfunction [3], increased risk of bone fractures due to decreased bone mineral density [4], hot flashes [5], reduced muscle strength, decreased lean body mass, increased fat mass, decreased insulin sensitivity [6] and potential harm to the cardiovascular system [7].

There are existing conflicting reports regarding the effect of ADT on cardiovascular disease. Saigal et al. showed that ADT is associated with cardiovascular mortality and may decrease survival in patients with low-risk disease [8].

Tsai et al. reported that ADT significantly increased mortality due to cardiovascular disease in patients who underwent radical prostatectomy for localized prostate cancer [9].

Nanda et al. found that ADT increased the risk of all-cause mortality in patients with a history of congestive heart failure or myocardial infarction, but this effect was not observed in patients without comorbidities or those with a single coronary artery risk factor [10].

However, a systematic review and meta-analysis found that ADT did not increase the risk of cardiovascular death and that ADT reduced prostate cancer-specific mortality and all-cause mortality [11]. A recent meta-analysis [12] suggested that ADT without estrogen increases the risk of thromboembolic events. A database study also showed that ADT significantly increased the risk of thromboembolic events [13]. A Swedish database study [14] also showed that the risk of thromboembolic events was increased in patients with prostate cancer. These studies focused on cardiovascular events. Teoh et al. [15] reported on 452 Chinese men with prostate cancer treated primarily with radical prostatectomy or radiotherapy and compared the risk of acute myocardial infarction (AMI) in patients who were given ADT with those who were not given any. The ADT group was associated with an increased risk of AMI (*P* = 0.004) when compared to the non-ADT group. The use of ADT (HR: 6.78, 95% confidence interval (CI): 1.31–35.05, *P* = 0.022) was a significant risk factor for AMI after multivariate Cox regression analysis. A retrospective study was then conducted by Teoh et al. [16] on 452 Chinese patients with prostate cancer. There were no statistically significant associations in the patients between administration of ADT and the development of ischemic stroke upon Kaplan–Meier analysis. However, upon multivariate Cox regression analysis, the use of ADT was a risk factor for ischemic stroke (HR: 3.32, 95% CI: 1.14–9.67, *P* = 0.028).

There are limited data about ischemic stroke and ADT in prostate cancer patients. The aim of our study was to investigate the safety of ADT as reflected by outcomes of ischemic stroke and mortality in a nationwide cohort study involving patients with prostate cancer using the Taiwan National Health Insurance Research Databases (NHIRD).

## Methods

### Database

A medical claims database, the NHIRD, was created for research and includes outpatient and inpatient claims data. The database includes information regarding each patient’s age, gender, diseases, prescription drugs, and medical expenditures. This study was a population-based retrospective cohort study. Our data were retrieved from the Longitudinal Health Insurance Database 2005, which includes data from 1,000,000 beneficiaries collected from 1997 to 2013. In this observational and analytical study, the database was derived from the NHIRD, and the data were deidentified secondary data that were released for research purposes. The study was reviewed and approved by the Institutional Review Board (IRB) of Kaohsiung Medical University Hospital. According to both the NHIRD and hospital regulations, informed consent from the patients was waived for this retrospective cohort study because the information was deidentified secondary data. All procedures were in accordance with the principles of the Declaration of Helsinki.

### Study population

We identified individuals older than 20 years of age who were newly diagnosed with prostate cancer (International Classification of Diseases, ninth revision, Clinical Modification [ICD-9-CM]: 185) between January 1, 2005, and December 31, 2012. Patients without certification for catastrophic illness were excluded. The index date was assigned as the date of diagnosis of prostate cancer.

We excluded patients who were diagnosed with other cancers before the index date and those who took any second-line ADT drugs (high dose bicalutamide, diethylstilbestrol, and medroxyprogesterone) within 180 days after the index date. Furthermore, patients who experienced an ischemic stroke or a transient ischemic stroke before the index date were excluded from our observational populations. We further excluded patients who had other cancer diagnoses, those who had received chemotherapy before the index date, those with a history of ischemic stroke or ADT exposure before the index date, and patients who had received second-line ADT.

### Drug use

Patients with prostate cancer who received at least one prescription for ADT within 6 months after the index date were defined as the ADT user group. Patients who did not receive at least one prescription for ADT within 6 months after the index date were defined as the ADT nonuser group. Each patient was followed until the first occurrence of one of the primary outcome measures (ischemic stroke or death), until they started receiving ADT if they were in the ADT user group, or until December 31, 2013.

### Comorbidities and medications

Patient comorbidities were identified according to diagnostic codes for each inpatient or outpatient diagnosis 1 year before the index date. Comorbidities included diabetes mellitus, hypertension, dyslipidemia, ischemic heart disease, congestive heart failure, chronic obstructive pulmonary disease (COPD), atrial fibrillation, thyroid disease, chronic kidney disease, and liver diseases. The demographic characteristics of the patients were also assessed at baseline.

The use of concomitant drugs was identified according to claimed prescriptions for one year before the index date. These drugs included angiotensin-converting enzyme inhibitors (ACEIs), angiotensin II receptor blockers (ARBs), diuretics, beta blockers, alpha blockers, calcium channel blockers (CCBs), 3-hydroxy-3-methylglutaryl-coenzyme A (HMG-CoA) reductase inhibitors (statins), antiplatelet drugs, nonsteroidal anti-inflammatory drugs (NSAIDs), vitamin K antagonists, and diabetes medications.

### Study endpoints

The composite primary endpoint was a combination of all-cause mortality or ischemic stroke. We also analyzed all-cause mortality and ischemic stroke as individual endpoints. For patients who visited the emergency department due to ischemic stroke, hospitalization due to ischemic stroke with a primary or secondary diagnosis code or hospitalization for carotid artery stent placement was defined as an ischemic stroke event.

### Statistical analysis

We aimed to describe the demographics including the ages, comorbidities, and concomitant medication use of prostate cancer patients with and without ADT exposure. For the baseline characteristics, continuous variables are presented as the means and standard deviations and were compared by Student’s t-tests. Discrete variables are presented as counts and percentages, and among patients with and without ADT exposure, these variables were compared by chi-square tests. Values of *P* < 0.05 were considered statistically significant in all statistical analyses.

We performed 1:1 propensity score (PS) matching on each sample. The variables including age groups and comorbidities were used to calculate the predicted probability from a logistic regression model. A Kaplan-Meier curve was used to estimate the differences between patients who did and did not use ADT after PS matching and was assessed by the log-rank test. A Cox proportional hazards model was used to assess the association of outcomes between patients with and without ADT exposure during the follow-up period. Models were adjusted for age group, comorbidities, and concomitant medication use. To assess the robustness of the outcomes, sensitivity analysis for ischemic stroke was performed in a competing risk model, and subgroup analysis was performed for ADT use (over one year and less than one year). All data management procedures and statistical analyses were performed using SAS software, version 9.4 (SAS Institute, Cary, NC, USA). The research was approved by the IRB of Kaohsiung Medical University Hospital (KMUHIRB-EXEMPT-20170011).

## Results

**Fig. 1:**
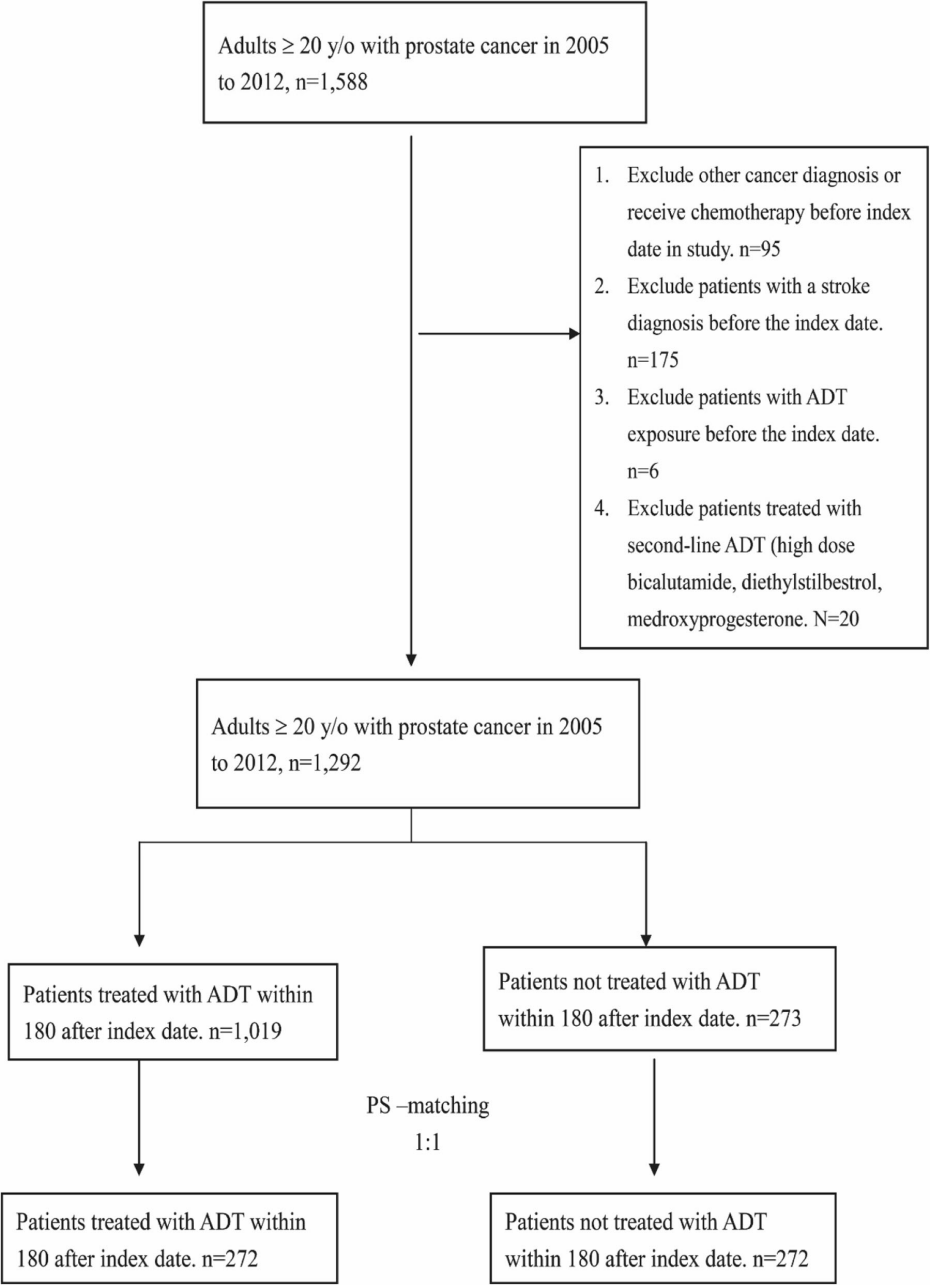
Study flowchart

We enrolled 1588 patients older than 20 years with prostate cancer. A total of 1292 patients met the inclusion and exclusion criteria. We performed 1:1 PS matching of these patients, and 272 patients were included for further analysis. Figure 1 shows a diagram of patient enrollment.

Table 1 shows the patient characteristics before and after PS matching. The mean patient age was approximately 70 years, and more than one-tenth of the patients were older than 80 years. The five most common comorbidities in order were hypertension, dyslipidemia, COPD, ischemic heart disease and diabetes. The three most common medications used were NSAIDs, alpha blockers and CCBs.

**Table 1:** Baseline characteristics of the prostate cancer patients after matching

	ADT Nonusers (*n* = 273)		ADT users (*n* = 1019)			PS Matching				
						ADT nonusers (n = 272)	ADT users (*n* = 272)			
Characteristic	n	%	n	%	*p*-value	n	%	n	%	*p*-value
Age, mean SD^a^	69.8	8.59	73.7	7.86	< 0.001	69.8	8.59	70.3	8.71	0.488
Age stratification					< 0.001					0.978
≤ 60	39	14.3	73	7.2		38	14.0	39	14.3	
61–70	98	35.9	247	24.2		98	36.0	96	35.3	
71–80	107	39.2	481	47.2		107	39.3	105	38.6	
> 80	29	10.6	218	21.4		29	10.7	32	11.8	
Comorbidity										
Diabetes mellitus	50	18.3	221	21.7	0.224	50	18.4	47	17.3	0.737
Ischemic heart disease	52	19.1	205	20.1	0.694	52	19.1	53	19.5	0.914
Congestive heart failure	16	5.9	76	7.5	0.362	16	5.9	16	5.9	1.000
COPD	59	21.6	249	24.4	0.331	59	21.7	53	19.5	0.525
Hypertension	139	50.9	565	55.5	0.182	138	50.74	131	48.2	0.548
Dyslipidemia	75	27.5	216	21.2	0.028	74	27.2	74	27.2	1.000
Thyroid disease	6	2.2	17	1.7	0.557	6	2.2	5	1.8	0.761
Atrial fibrillation	4	1.5	19	1.9	0.658	4	1.5	2	0.7	0.412
Liver disease	32	11.7	117	11.5	0.912	32	11.7	32	11.7	1.000
Renal disease	14	5.1	72	7.1	0.254	14	5.2	9	3.3	0.287
Comedications										
ACEIs	34	12.5	158	15.5	0.281	34	12.5	38	14.0	0.613
ARBs	34	12.5	163	16.0	0.148	33	12.1	43	15.8	0.216
Beta blockers	76	27.8	261	25.6	0.457	75	27.6	70	25.7	0.628
CCBs	108	39.6	419	41.1	0.642	107	39.3	100	36.8	0.537
Alpha blockers	80	29.3	381	37.4	0.013	80	29.4	102	37.5	0.046
NSAIDs	231	84.6	837	82.1	0.337	230	84.6	227	83.5	0.726
Statins	46	16.9	122	12.0	0.033	45	16.5	37	13.6	0.338
Antiplatelet drugs	52	19.1	207	20.3	0.643	52	19.1	49	18.0	0.741
Anticoagulants	2	0.7	12	1.2	0.528	2	0.7	5	1.8	0.254
Antidiabetic drugs	38	13.9	183	18.0	0.116	38	14.0	44	16.2	0.472

*SD* standard deviation

*ACEIs* angiotensin-converting enzyme inhibitors

*ARBs* angiotensin receptor blockers, *CCBs* calcium channel blocker, *NSAIDs* nonsteroidal anti-inflammatory drugs

^a^Student’s t-test

Table 2 shows the outcomes of ADT users and nonusers with prostate cancer after PS matching. The primary composite outcome was the time to any cause of death or to ischemic stroke. There was no significant difference in primary composite outcome among ADT users and nonusers with prostate cancer. In terms of any cause of mortality, prostate cancer patients who received ADT had a higher mortality rate than those who did not receive ADT, and the adjusted hazard ratio was 1.907 (95% CI: 1.278–2.844; *P* = 0.0016) after adjusting for age, diabetes mellitus, ischemic heart disease, congestive heart failure, COPD, hypertension, dyslipidemia, thyroid disease, atrial fibrillation, liver disease, renal disease and comedication, as shown in Table 1. The results showed that patients treated with ADT during follow-up did not have a significantly higher risk of ischemic stroke than those not treated with ADT (crude HR: 1.147, *P* = 0.3451; adjusted HR: 0.812, *P* = 0.3291).

**Table 2:** Cox proportional hazard models comparing ADT users and nonusers with prostate cancer after matching

Outcomes	Before Matching					Propensity Score Matching				
	ADT Nonuser	ADT user				ADT Nonuser	ADT user	Adjusted HR^a^	95% CI	*p*-value
	Event	Event	Adjusted HR^a^	95% CI	*p*-value	Event	Event			
Primary outcome	82 (30.0)	499 (49.87)	1.332	1.051–1.687	0.0177*	82 (30.2)	114 (41.9)	1.192	0.885–1.605	0.2471
Any cause of death	37 (13.6)	375 (36.8)	2.107	1.499–2.900	< 0.0001*	37 (13.6)	85 (31.3)	1.907	1.278–2.844	0.0016*
Ischemic stroke	49 (18.0)	203 (19.9)	0.973	0.709–1.334	0.8640	49 (18.0)	45 (16.5)	0.812	0.534–1.234	0.3291

*HR* hazard ratio, *CI* confidence interval

* *p*-value < 0.05

^a^ adjusted variables included age group, comorbidities and comedications

In subgroup analysis, the patients who used ADT over one year did not have a significantly increased risk of ischemic stroke than the ADT nonuser patients (adjusted HR: 1.132, *P* = 0.5745); there was also no significant difference in ischemic stroke risk between patients who used ADT for less than a year and those who did not use ADT (adjusted HR: 1.217 (*p* = 0.2311)). Furthermore, we also repeated the analysis for ischemic stroke in a competing risk model (adjusted HR: 0.759, *P* = 0.2050), which also showed that ADT use did not significantly increase the risk of ischemic stroke.

**Fig. 2:**
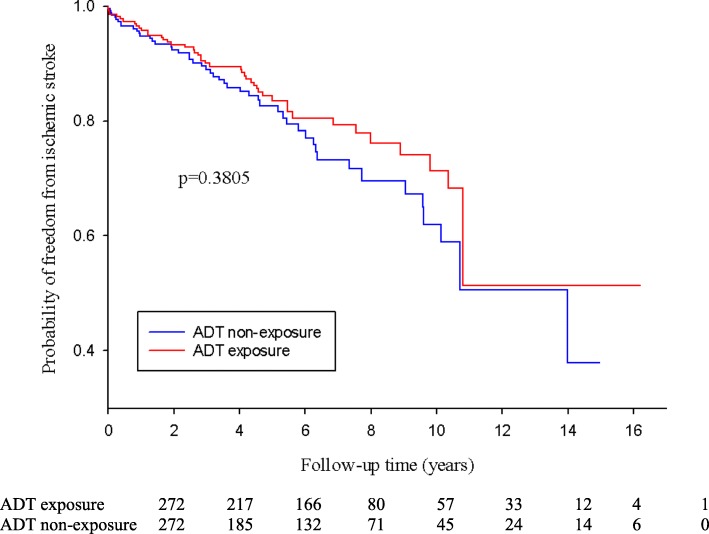
Probability of freedom from ischemic stroke following propensity score matching stratified by ADT exposure and non-exposure

Figure 2 shows the probability of freedom from ischemic stroke after propensity score matching stratified by ADT users and nonusers. There was no significant difference in ischemic stroke between ADT users and nonusers. (*P* = 0.3805).

## Discussion

In prostate cancer patients treated with ADT, the severity conferred by endocrine treatment was greater than that of the other treatments. These prostate cancer patients with more advanced disease may experience a poor prognosis and high mortality.

Albertsen et al. [17] performed six phase 3 prospective randomized trials to show cardiovascular morbidity following initiation of gonadotropin-releasing hormone agonists compared with an antagonist. The risk of cardiac events within 1 year of initiating therapy was lower in patients received gonadotropin-releasing hormone antagonists compared with gonadotropin-releasing hormone agonists (HR: 0.44, 95% CI: 0.26–0.74, *P* = 0.002).

Teoh et al. [18] found that surgical castration was associated with an increased risk of cardiovascular thrombotic events when compared to gonadotropin-releasing hormone agonists. After multivariate Cox regression analysis, age, hyperlipidemia, and surgical castration were significant risk factors of cardiovascular thrombotic events. Chung et al. [19] also used the NHIRD and found that there was no significant difference in the risk of stroke in patients with prostate cancer who did and did not receive ADT in Taiwan, after adjusting for potential confounders. Some physiological and genetic differences have been observed in different ethnicities. The UGT2B gene and androgen receptor genetic polymorphisms, namely, the CAG repeat length polymorphism, may possibly lead to differences in the cardiovascular risk between Asians and Caucasians. Asians have longer CAG repeat lengths than Caucasians, and testosterone suppression with ADT may lead to higher cardiovascular risk in Asians than in Caucasians, although we need more evidence to support this hypothesis [20].

Prostate cancer patients who received ADT seemed to be at an increased risk of having thromboembolic events, including arterial embolism, pulmonary embolism or deep venous thrombosis. A recent meta-analysis including 10 studies found that ADT increased the risk of thromboembolic events with a risk ratio of 1.43 (95% CI: 1.15–1.77, *P* = 0.001) [12]. Another database study analyzed 58,466 patients treated with ADT [13] and found that ADT was associated with an increased risk of thromboembolic events (adjusted hazard ratio = 1.56; 95% CI: 1.50–1.61; *P* < 0.0001).

A Swedish database study analyzed 30,642 men with prostate cancer on endocrine therapy and found increased risks of deep vein thrombosis (standardized incidence ratio (SIR) = 2.48, 95% CI = 2.25–2.73) and pulmonary embolism (SIR = 1.95, 95% CI = 1.81–2.15) but did not increase in arterial embolism (SIR = 1.00, 95% CI = 0.82–1.20) [14].

Malignant disease is associated with the risk of thromboembolic disease and has long been recognized in clinical practice. Previous studies have reported this risk of thromboembolism in patients with prostate cancer, but these studies did not focus on ischemic stroke [21–23]. Other malignant diseases, such as breast cancer, malignant gliomas and ovarian cancer, are also associated with a high risk of thromboembolic events affecting 1 to 28% of patients [22].

Graus et al. [24] conducted an autopsy study to assess the prevalence of pathological findings of cerebrovascular disease in cancer patients and found that 14.6% of patients had pathologic evidence of cerebrovascular disease, and 7.4% had clinical symptoms of cerebrovascular disease. The risk factors for cerebrovascular disease were obscured by pathophysiologic factors associated with malignancy, including direct effects of the malignancy, hypercoagulable state, susceptibility to infections, treatment medication and the process of treatment or diagnosis. Cestari et al. [25] used a registry database to evaluate the incidence and type of strokes in cancer patients and showed that the three most common primary cancers were lung cancer (30%), brain cancer (9%) and prostate cancer (9%). The most common cause of stroke was embolic stroke, occurring in 52 (54%) patients. Forty-four (46%) patients had nonembolic stroke. The mechanism of stroke was partially due to hypercoagulability, and atherosclerosis only explained 22% of strokes in cancer patients. Outcome s were primarily decided by the underlying cancer stage.

Bosco et al. [26] included 8 studies in a meta-analysis and found a positive association between ADT and the risk of cardiovascular disease. In their meta-analysis, most studies focused on myocardial infarction, and one study focused on ADT and ischemic stroke. Azoulay et al. [27] used the General Practice Research Database, a primary care database from the United Kingdom, to conduct a nested case-control analysis and found that ADT increased the risk of stroke or transient ischemic attacks in patients with prostate cancer. In contrast to their study, our study found that ADT was not associated with the risk of ischemic stroke in prostate cancer. There are some differences between their study and our study. First, although both retrospective studies used a database, our study design was a cohort study, while Azoulay et al. used nested case-control analysis. In the presence of competing risks, the use of a nested case-control design results in greater bias compared to the use of a cohort design whether treatment occurs at baseline or varies over time and there is a single outcome. The cohort design has greater precision and a lower mean squared error in all scenarios [28]. Second, we used PS matching and adjusted for more cardiovascular medications. Third, their definition of stroke included hemorrhagic stroke because the stroke subtype (ischemic vs hemorrhagic) cannot be differentiated from the General Practice Research Database in the United Kingdom. Fourth, they excluded patients with evidence of metastases at diagnosis. We enrolled patients with all stages of prostate cancer.

Our population-based cohort study has some advantages. First, this was a large population-based cohort of patients with prostate cancer who were followed for up to 9 years. Second, the National Health Insurance program in Taiwan is compulsory for all citizens and covers almost all services that can be provided by a healthcare system. Third, we enrolled all patients with prostate cancer who were treated with ADT. Fourth, in Taiwan, patients with malignant diseases have catastrophic illness certification and do not need to pay a copayment for outpatient or inpatient care. Their applications are formally reviewed by more than two physicians and have a low probability of misdiagnosis. The prescriptions in Taiwan are also written by specialists (urologists or oncologists); thus, patients have a low probability of being misclassified as ADT users or ADT nonusers.

There are some limitations to our study. The NHIRD lacks some confounders, such as body mass index, cancer stage and smoking status. Therefore, we were unable to adjust for these confounders. This study evaluated the association of ADT and ischemic stroke in all prostate cancer patients regardless of the stage of disease. Second, there may be some asymptomatic ischemic stroke patients who were not diagnosed. These patients did not receive comprehensive routine brain magnetic resonance imaging examinations; thus, small stroke lesions may have been omitted. It is also possible that a rapidly massive fatal ischemic stroke in a prostate cancer patient with advanced disease could have been interpreted as the fatal end-stage of prostate cancer and therefore was not classified as our interest outcome of ischemic stroke. However, we believe that only a small number of cases may have been misclassified in this condition. Babiker et al. [29] suggested that prostasomes, submicron secretory granules from the prostate gland, evoke blood coagulation in prostate cancer and result in thromboembolic disease. Other studies [30, 31] have also shown a link between testosterone and enhancement of fibrinolytic inhibition via increased synthesis of the plasminogen activator inhibitor plasminogen activator inhibitor-1, but our study found that the risk of ischemic stroke did not significantly increase in prostate cancer patients who received ADT. We may need a prospective study with a large sample size to assess the association between ischemic stroke and ADT, and whether there is a different thromboembolic mechanism between myocardial infarction and ischemic stroke in prostate cancer patients receiving ADT needs to be further investigated.

## Conclusions

In this nationwide population-based study, we compared the risk of ischemic stroke between patients with prostate cancer who were and were not treated with ADT. Our study showed that patients with prostate cancer who received ADT did not have an increased risk of ischemic stroke, but they did have an increased risk of mortality, which may result from confounding by indication.

## Data Availability

The data that support the findings of this study are available from the NHIRD, but restrictions apply to the availability of these data, which were used under license for the current study and so are not publicly available. Data are, however, available from the authors upon reasonable request and with permission from the NHIRD.

## Abbreviations

ACEIs: Angiotensin-converting enzyme inhibitors
ADT: Androgen deprivation therapy
ARBs: Angiotensin II receptor blockers
CCBs: Calcium channel blockers
CI: Confidence interval
COPD: Chronic obstructive pulmonary disease
HR: Hazard ratio
NHIRD: National Health Insurance Research Databases
NSAIDs: Nonsteroidal anti-inflammatory drugs
PS: Propensity score
SIR: Standardized incidence ratio
